# Telehealth Use Among Older Adults Receiving Home- and Community-Based Services: Cross-Sectional Analysis Using the National Core Indicators–Aging and Disabilities Survey

**DOI:** 10.2196/77522

**Published:** 2026-06-02

**Authors:** Dana P Urbanski, Romil R Parikh, Benjamin W Langworthy, Jack M Wolf, Chanee D Fabius, Janette Dill, Eric Jutkowitz, Tetyana P Shippee

**Affiliations:** 1 College of Arts and Sciences, Indiana University Bloomington, 2631 E Discovery Pkwy, Bloomington, IN, 47408, United States, 1 440-523-0350; 2 Division of Health Policy & Management, School of Public Health, University of Minnesota, Minneapolis, MN, United States; 3 Division of Biostatistics & Health Data Science, School of Public Health, University of Minnesota, Minneapolis, MN, United States; 4 Department of Health Policy and Management, Bloomberg School of Public Health, Johns Hopkins University, Baltimore, MD, United States; 5 Department of Health Services, Policy & Practice, School of Public Health, Brown University, Providence, RI, United States; 6 Center of Innovation in Long Term Services and Supports, Providence VA Medical Center, Providence, RI, United States; 7 Evidence Synthesis Program Center, Providence VA Medical Center, Providence, RI, United States

**Keywords:** telehealth, older adults, home- and community-based services, aging in place, internet access

## Abstract

**Background:**

Telehealth was essential for maintaining care continuity during the COVID-19 pandemic, leading to its rapid adoption across the United States. Telehealth has been heralded as a strategy for improving health care access and reducing health disparities, especially for community-dwelling older adults who face significant barriers to in-person care. However, data on telehealth use among socially and financially vulnerable older adults are limited, and little is known about characteristics associated with telehealth use in this population.

**Objective:**

Guided by the Systems Engineering Initiative for Patient Safety (SEIPS) 3.0 framework, this study examined factors associated with postpandemic telehealth use among older adults living at home and receiving publicly funded home- and community-based services (HCBS), considering HCBS receipt as an indicator of social and financial vulnerability.

**Methods:**

This cross-sectional study included older adults aged 65 years or older living at home with available telehealth use data who participated in the 2021‐2022 survey wave of the National Core Indicators-Aging and Disabilities Adult Consumer Survey. We used complete-case multivariable logistic regression, adjusting for sociodemographic and health-related factors with state-level random intercepts, to examine associations between telehealth use and covariates of interest (age, sex, race/ethnicity, zip code, rural-urban commuting area code, internet access, self-perceived overall health, medical transportation access, living alone, number of known non–Alzheimer disease and related dementias [ADRD] diagnoses, known ADRD diagnosis, and HCBS program/payer type). Based on the regression results, we estimated bivariate associations between internet access and key sociodemographic variables (age, sex, race/ethnicity, and zip code rural-urban commuting area) using the Pearson chi-square test. Findings were organized and interpreted through the SEIPS 3.0 framework.

**Results:**

Of the 3680 participants, 1467 (40%) were telehealth users and 2213 (60%) were nonusers. Significantly lower odds of telehealth were observed for older adults in older age groups, males, Black individuals, those living in nonmetropolitan areas, and recipients of Older Americans Act services (odds ratios [OR] between 0.66 and 0.80). Individuals with more than one known non-ADRD diagnosis (OR 1.49, 95% CI 1.02‐2.17) and those with an ADRD diagnosis (OR 1.33, 95% CI 1.07‐1.66) had higher odds of telehealth use. Internet access was strongly associated with telehealth use (OR 2.51, 95% CI 2.15‐2.92). Follow-up bivariate analyses between internet access and sociodemographic characteristics revealed that those of younger age, females, and White individuals had higher levels of internet access.

**Conclusions:**

Differences in telehealth use among older HCBS recipients are associated with multiple individual, technological, and organizational factors. Interpreted through the SEIPS 3.0 framework, these findings underscore the importance of viewing telehealth use as the outcome of multiple features of the health care system. Future research should clarify the mechanisms driving variation in telehealth use to identify and address barriers to telehealth adoption among vulnerable older adults.

## Introduction

During the COVID-19 pandemic, telehealth emerged as a key strategy for maintaining continuity of care amid lockdowns and social distancing measures [1-3]. Consequently, American patients and providers alike became more accepting of telehealth [4-7], contributing to a rapid increase in its use across the United States [8-10]. This surge in telehealth activity—defined as the use of telecommunications technology to provide health care remotely [11]—has since facilitated the continued integration and expansion of telehealth in United States health care delivery [9,12,13]. As its use has grown, telehealth has been increasingly viewed as a means of improving health care access and addressing potential disparities in health outcomes [14-17]. Proponents of telehealth emphasize its potential for significantly reducing cost and access barriers to care, especially for those who are medically vulnerable, geographically isolated, and socially marginalized [14-17]. For this reason, telehealth is seen as a particularly promising approach for improving access to both routine geriatric care and specialty care for chronic and complex conditions among community-dwelling older adults [17-19]. Across the United States, many older adults lack access to in-person care due to health and mobility-related challenges, combined with structural and sociodemographic factors that drive health disparities, including those related to race, ethnicity, socioeconomic status, and rurality [20-26]. For these individuals in particular, telehealth may be a promising strategy for improving access to health care and potentially reducing disparities [27].

Despite this promise, data on the demographics and characteristics of older telehealth users are limited, and it remains unclear whether telehealth improves access to care for older Americans. Available data indicate that, overall, telehealth use is relatively low among older adults, with only about one-third using telehealth [28,29]. Importantly, telehealth serves a wide range of functions beyond the management of diagnosed conditions. It is used for screening/triage, routine monitoring, medication review, mental health services, and supportive or social follow-up, services that are highly relevant for many older adults [1,6,19]. The fact that telehealth use remains relatively low among older adults despite these broad potential applications underscores the need to better understand the factors that shape telehealth use.

Broad, overarching analyses on telehealth use among older adults may conceal underlying differences in telehealth access and use driven by individual factors, such as age, sex, health status, and preference, as well as organizational and environmental factors, including health care systems, geographic location, and access to infrastructure that supports telehealth use (eg, broadband internet). These sets of factors, and their interactions, likely influence telehealth use among older adults. Recent data show that among American adults broadly, older age, male sex, rurality, and lack of internet access are negatively associated with telehealth use [30-32]. An emerging literature suggests these factors are also associated with telehealth use among older adults; however, older adults are also disproportionately affected by additional barriers to telehealth use, including inexperience with technology, lack of social support for managing telehealth technology, physical disability, and dementia [33-35]. More research is needed to identify the factors associated with telehealth use among older adults, with particular attention to conditions that contribute to potential disparities in access and use.

One such population is older adults who receive publicly funded home- and community-based services (HCBS). This group is particularly important to examine with respect to telehealth use because their substantial care and social needs make them likely to benefit from remote care while at the same time leaving them vulnerable to barriers that may limit telehealth use. HCBS are a broad range of services (such as personal care, homemaker, meal delivery, adult day care, and transportation services) designed for older adults with physical and/or cognitive functional impairments to receive care in their own homes or communities rather than in nursing homes or other institutional settings [36].

In the United States, HCBS are delivered through several publicly funded program types that vary in their structure, degree of integration with the formal health care system, and scope of benefits [37]. Major HCBS programs include Medicaid 1915 (c) waiver-funded HCBS, services authorized under the Older Americans Act (OAA) and administered by local Area Agencies on Aging (AAAs), managed long-term services and supports (MLTSS), and fully integrated care models such as the Program of All-Inclusive Care for the Elderly (PACE) [36]. Medicaid-funded HCBS programs, MLTSS, and PACE are directly connected to health care financing and typically include coverage of medical services alongside long-term services and supports. In contrast, OAA-funded services provide supportive services—such as meals, transportation, and referrals—rather than direct medical care.

More than 4.5 million older adults in the United States use HCBS [37]. This group represents a high-need population with complex health care needs, and their receipt of publicly funded HCBS serves as an indicator of their financial and social vulnerability [36]. At the same time, HCBS recipients are insured and already integrated into formal health care delivery systems, enabling examination of telehealth use among vulnerable older adults without the confounding factor of basic access to health insurance.

This study examines telehealth use among older adults who receive publicly funded HCBS and live in their own or family home. We approach this question using a human factors lens, asking not simply whether older adults adopt telehealth but how system elements at the individual, organizational, and environmental levels are associated with its use. To this end, we use the Systems Engineering Initiative for Patient Safety (SEIPS) 3.0 framework [38], a human factors model that conceptualizes health care outcomes, such as telehealth use, as emerging from interactions among persons, tools and technology, environments, and organizations. In this study, SEIPS is used as an organizing framework to classify measured variables reflecting person characteristics (eg, age, sex, and health status), tools and technology (eg, internet access), environmental context (eg, rurality), and organizational features (eg, HCBS program type). This framework is well suited for understanding telehealth use among community-dwelling older adults receiving HCBS, whose social and financial vulnerability warrants careful attention to the individual, environmental, technological, and organizational factors that shape their interactions with the health care system.

We examine patterns of postpandemic telehealth use among older HCBS recipients using data from the National Core Indicators–Aging and Disabilities (NCI-AD) Adult Consumer Survey. It is important to note that the NCI-AD dataset reflects real-world reported telehealth use and does not indicate whether participants were explicitly offered telehealth by their providers or payers. This study therefore focuses on patterns of reported use among individuals with potential access through their existing service networks, providing insight into which subpopulations of older adults are—or are not—being reached by telehealth in practice. These findings can inform efforts to identify subpopulations of older adults who are underrepresented among telehealth users and the systems-level barriers that may limit their engagement with telehealth. Such data are essential for beginning to identify opportunities to expand telehealth among medically vulnerable older adults and ensure telehealth reaches its full potential for improving access to care.

## Methods

### Data Source and Study Sample

This study used cross-sectional data from the 2021‐2022 cycle of the NCI-AD Adult Consumer Survey, which aims to evaluate and monitor the effectiveness of publicly funded HCBS and other long-term care services and supports for older adults and individuals with disabilities. State participation in the NCI-AD is optional. Participating states administer NCI-AD to a representative sample of HCBS recipients each year. The NCI-AD survey consists of 2 sections. The first section gathers background information such as demographics, health conditions, and service use, typically sourced from state administrative records. The second section is a direct-conversation consumer interview and survey that collects person-centered data on the HCBS recipient’s health, functional abilities, and care experiences. If an HCBS recipient cannot participate, a close proxy may complete portions of the survey that collect objective, observable information.

**Figure 1.**
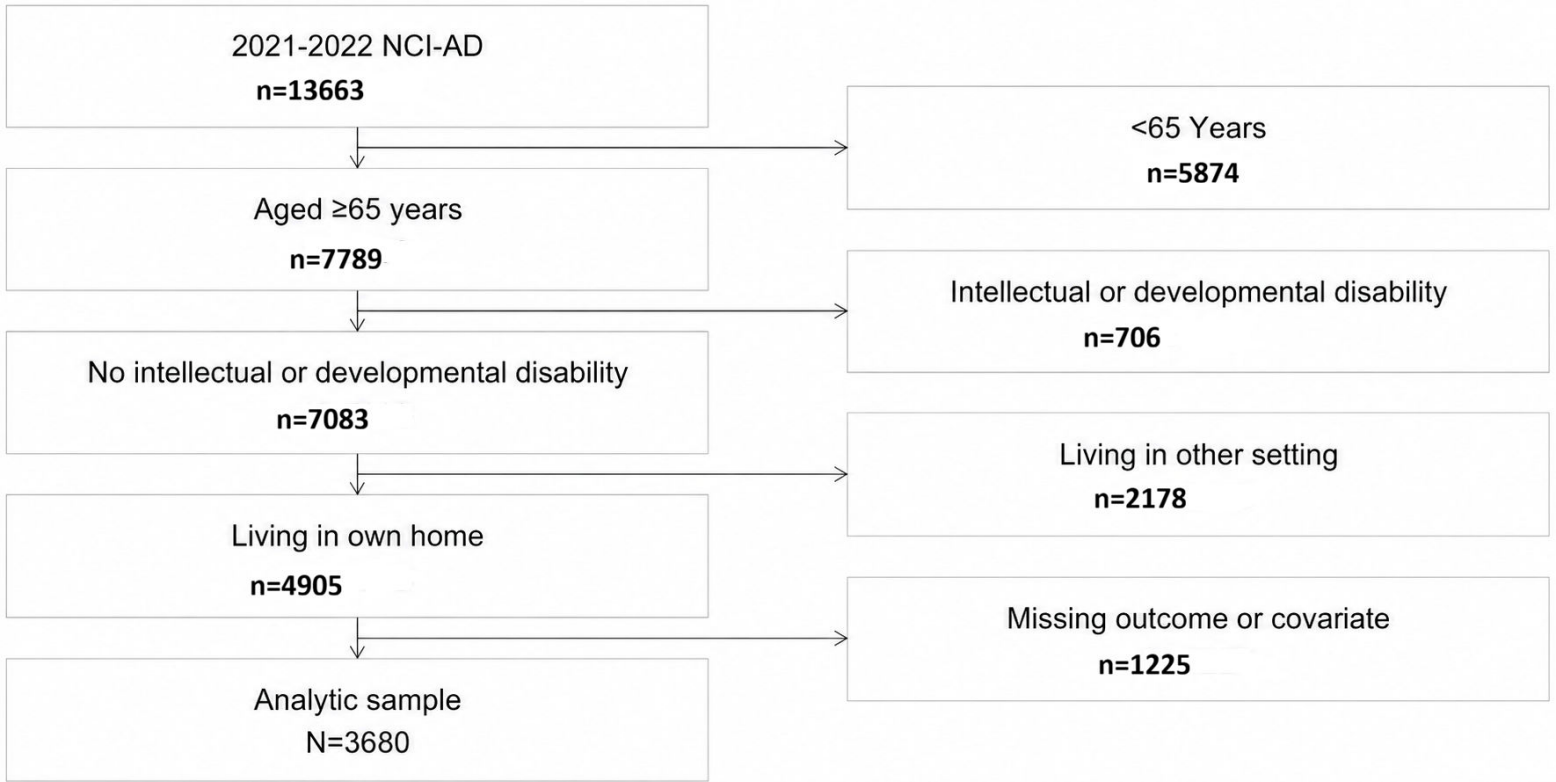
Flow diagram for construction of the analytic sample. NCI-AD: National Core Indicators–Aging and Disabilities.

We focused on the 2021‐2022 wave of the NCI-AD Adult Consumer Survey because it provided the most recent data and reflects telehealth use during the postpandemic period when telehealth use has largely stabilized [39]. The 2021‐2022 survey included 13,663 participants from 15 states (Colorado, Delaware, Georgia, Indiana, Kansas, Michigan, Minnesota, Missouri, New Jersey, Ohio, Oklahoma, Tennessee, Texas, Washington, and Wisconsin). For the present analysis, we focused on recipients of publicly funded HCBS aged 65 years and older, who lived in their own or family home, did not have an intellectual or developmental disability, did not receive a nursing facility waiver, had nonmissing telehealth use data, and had complete covariate data (described below), resulting in a final analytic sample of 3680 participants. Figure 1 shows the detailed participant flow diagram. The percentage of missing data was below 10% for all individual items, including telehealth use. In total, 85% of participants with nonmissing telehealth data had complete data and were included in the complete-case regression analyses.

### Telehealth Use

The primary dependent variable of interest, telehealth use, was determined by an interview question that asked participants to report if they had ever communicated with health professionals via videoconferencing or telehealth. Possible responses were “yes,” “no,” “don’t know,” or “unclear/refused/no response.” All “yes” responses were categorized as telehealth users, and “no” responses were considered nontelehealth users. All remaining responses were categorized as missing and excluded from the analyses.

### Covariates

Covariates were selected based on the broader health care and telehealth literature and aligned with the SEIPS 3.0 human factors framework. As described previously, SEIPS 3.0 conceptualizes health care outcomes, such as telehealth use, as emerging from interactions among persons, tools and technology, environments, and organizations. Consistent with this framework, covariates were grouped according to these elements.

Person factors reflect the individual characteristics, capabilities, and experiences that shape how individuals interact with and navigate health care systems. This category included demographic characteristics (age, sex, and race/ethnicity); self-perceived overall health; lives alone; number of known non–Alzheimer disease and related dementias (ADRD) diagnoses; known ADRD diagnosis; and response type (self vs proxy response). We classified age into a categorical measure describing age group, including 65‐74 years, 75‐84 years, and ≥85 years; sex was categorized as female or male (no participants selected a sex other than male or female); and race and ethnicity were grouped as Black or African American, Hispanic or Latino, White, and multiple race/ethnicity. Age, sex, and race/ethnicity were populated from the background administrative section of the NCI-AD.

Self-perceived overall health was determined through the NCI-AD interview, with possible responses of poor, fair, good, very good, and excellent. The covariate “lives alone” indicates whether the participant lives independently, with participant interview responses categorized as “yes” for those living alone and “no” for those residing with others. The number of known non-ADRD diagnoses refers to the count of medical conditions excluding ADRD (including brain injury, cancer, chronic obstructive pulmonary disease, stroke, diabetes, hypertension or high blood pressure, heart disease, physical disability, and mental health diagnoses). ADRD was accounted for separately under the covariate known ADRD diagnosis, which included all individuals with a recorded ADRD diagnosis. ADRD and non-ADRD diagnoses were obtained from state administrative records when available or from participant reports, both linked to the NCI-AD survey dataset. Administrative records included provider-documented diagnoses (eg, *ICD-10* [*International Statistical Classification of Diseases, 10th Revision*] diagnosis codes or I4200 or I4800 Minimum Data Set fields). Finally, the covariate *proxy response* refers to whether the individual completed the interview independently through self-response or if a close proxy provided the answers on their behalf.

Tools and technology are the physical and digital resources that enable or constrain how individuals engage with health care systems. Within this framework, we examined internet access, which represents a foundational technological tool that enables older adults to interact with the health care system through telehealth. In the NCI-AD survey, internet access was defined as the availability of internet service at home, a coffee shop, or somewhere else the participant spends time during the day. This item was assessed via direct interview, with participant interview responses categorized as “no or sometimes” and “yes.”

Environmental factors encompass the external conditions that shape how individuals interact with health care systems. These factors include geography, built environment, and community resources, which can either support or constrain care delivery and access. In this study, environmental factors included zip code rural-urban commuting area (RUCA) classification and medical transportation access. Zip code RUCA classification was used to approximate participants’ geographic environment and degree of rurality and was classified into 4 levels, including metropolitan (areas with a population of 50,000 or more), micropolitan (areas with a population of between 10,000 and 49,999), small town (areas with a population under 10,000 that have commuting ties to an urban area), and rural (areas with no commuting ties to any urban area). Medical transportation access was assessed by the interview question, “Do you have transportation to get to medical appointments when you need to?” Participants could respond with “no,” “sometimes,” or “yes.” Responses of “no” and “sometimes” were grouped to represent participants with at least some transportation barriers, while “yes” responses were retained as a separate category to represent those who have consistent access to transportation for in-person care. This variable was conceptualized as an environmental factor capturing the physical and community-level conditions that influence an individual’s ability to access health care.

Organizational factors refer to the health care delivery organizations in which the patient journey occurs, characterized by workflows, policies, resource allocation, and interorganizational coordination that may influence patients’ telehealth use. In this study, organizational factors were included as HCBS program type, which refers to the HCBS program administering or providing services as indicated by administrative records, classified into 3 categories, including PACE/MLTSS, OAA, and Medicaid. We combined PACE and MLTSS due to the small number of PACE participants (PACE: n=55; MLTSS: n=1259) in our sample and the shared characteristics of both programs. Both PACE and MLTSS operate under a capitated payment structure and are designed to serve older adults requiring long-term care. Since approximately 90% of our analytic sample reported receiving Medicare, consistent with United States census data [40], we focused on other payment types due to the low variance in Medicare enrollment.

### Statistical Analyses

Descriptive statistics were used to summarize the analytic sample of participants with complete data for all included covariates. Bivariate associations between covariates and telehealth use were assessed using chi-square tests and 2-sample 2-tailed *t* tests. We then estimated the adjusted associations between telehealth use and each of the covariates described using complete-case multivariable logistic regression, in which each coefficient represents the association of a given covariate with telehealth use while holding all other covariates constant. State was included as a random effect in the logistic regression model to account for potential variation in telehealth use across different states. To evaluate the impact of missing data, we conducted a sensitivity analysis using multiple imputation by chained equations [41,42], averaging results across 10 imputed datasets with no missingness (n=4786 per dataset).

Given the strong association between internet access and telehealth use in the complete-case regression analysis, we performed post hoc bivariate analyses on the analytic sample from the complete-case regression model to examine sociodemographic factors that may influence internet access as a potential precursor to telehealth use. We conducted bivariate analyses using chi-square tests to test the independence of internet access and the following factors: age, sex, race/ethnicity, and zip code RUCA classification.

All analyses were performed using R (version 4.4.2; R Project for Statistical Computing). *P*<.05 was considered statistically significant.

### Ethical Considerations

This study used deidentified secondary data from the 2021-2022 NCI-AD Adult Consumer Survey. The study was submitted to the University of Minnesota Institutional Review Board and was deemed exempt from human subjects review (STUDY00010799). Because the present analysis used deidentified secondary data, additional informed consent from participants was not required. No compensation was provided to participants for this secondary analysis.

## Results

### Participant Characteristics

Table 1 presents the complete characteristics of the analytic sample. Percentages shown in Table 1 summarize the characteristics of the analytic sample and are not intended as population estimates; therefore, 95% CIs were not calculated for these descriptive values. The analytic sample included 3680 participants, with 1467 out of 3680 (40%) reporting ever using telehealth and 2213 out of 3680 (60%) reporting never using telehealth. Most participants were female (2667/3680, 72%) and most resided in metropolitan areas (2660/3680, 72%), while smaller proportions lived in micropolitan (528/3680, 14%), rural (219/3680, 6%), or small-town (273/3680, 7.4%) settings. The sample was racially diverse, with 1929 out of 3680 (52%) identifying as White, 858 out of 3680 (23%) as Black, 443 out of 3680 (12%) as Hispanic or Latino, and 450 out of 3680 (12%) as multiple racial/ethnic identities. Overall, 2144 out of 3680 (58%) reported having internet access, while 1536 out of 3680 (42%) reported no or intermittent access. In terms of health characteristics, 3161 out of 3680 (86%) participants did not have a documented ADRD diagnosis, and 2988 out of 3680 (81%) participants had more than one known non-ADRD diagnosis. Self-perception of health varied; most participants rated their overall health as fair (1519/3680, 41%) or good (1176/3680, 32%). About half of participants lived with at least one other person (1937/3680, 53%), and most reported having access to medical transportation (3395/3680, 92%). HCBS program enrollment was distributed among Medicaid (1630/3680, 44%), OAA programs (736/3680, 20%), and PACE/MLTSS (1314/3680, 36%).

Older HCBS recipients who were younger, female, had multiple diagnoses, lived with others, and had a known ADRD diagnosis had higher rates of telehealth use. In contrast, Black individuals and those from nonmetropolitan areas had lower rates of telehealth use compared to their counterparts. Participants with lower perceptions of their overall health tended to be more likely to use telehealth, though this relationship was not significant. Internet access showed a clear pattern with telehealth use: 49% (1047/2144) of those who reported having internet access were telehealth users, while only 27% (420/1536) of those with no or intermittent access reported using telehealth.

**Table 1.** Sample characteristics and predictor frequencies from National Core Indicators–Aging and Disabilities (NCI-AD) 2021‐2022 by telehealth use. Participants are publicly funded home- and community-based services recipients aged 65 years and older without intellectual or developmental disabilities.

Characteristic	Overall (N=3680)	Telehealth use		*P* value^a^
		No, n=2213 (60)	Yes, n=1467 (40)	
Age (years), n (%)				<.001
65-74	1783 (48)	990 (56)	793 (44)	
75-84	1238 (34)	792 (64)	446 (36)	
≥85	659 (18)	431 (65)	228 (35)	
Sex, n (%)				.003
Female	2667 (72)	1564 (59)	1103 (41)	
Male	1013 (28)	649 (64)	364 (36)	
Race/ethnicity, n (%)				<.001
White	1929 (52)	1172 (61)	757 (39)	
Black	858 (23)	565 (66)	293 (34)	
Hispanic or Latino	443 (12)	223 (50)	220 (50)	
Multiple race/ethnicity	450 (12)	253 (56)	197 (44)	
Zip code RUCA^b^, n (%)				<.001
Metropolitan	2660 (72)	1523 (57)	1137 (43)	
Micropolitan	528 (14)	350 (66)	178 (34)	
Small town	273 (7.4)	189 (69)	84 (31)	
Rural	219 (6.0)	151 (69)	68 (31)	
Internet access, n (%)				<.001
No or sometimes	1536 (42)	1116 (73)	420 (27)	
Yes	2144 (58)	1097 (51)	1047 (49)	
Self-perceived overall health, n (%)				.06
Poor	604 (16)	341 (56)	263 (44)	
Fair	1519 (41)	904 (60)	615 (40)	
Good	1176 (32)	737 (63)	439 (37)	
Very good	306 (8.3)	191 (62)	115 (38)	
Excellent	75 (2.0)	40 (53)	35 (47)	
Medical transportation access, n (%)				.10
No or sometimes	285 (7.7)	185 (65)	100 (35)	
Yes	3395 (92)	2028 (60)	1367 (40)	
Lives alone, n (%)				.008
No	1937 (53)	1125 (58)	812 (42)	
Yes	1743 (47)	1088 (62)	655 (38)	
Number of known non-ADRD^c^ diagnoses				<.001
Mean (SD)	3.03 (1.62)	2.88 (1.60)	3.26 (1.63)	
Median (IQR)	3 (2-4)	3 (2-4)	3 (2-4)	
Number of known non-ADRD diagnoses (categorical), n (%)				<.001
0	166 (4.5)	121 (73)	45 (27)	
1	526 (14)	351 (67)	175 (33)	
More than 1	2988 (81)	1741 (58)	1247 (42)	
Known ADRD diagnosis, n (%)				.001
No	3161 (86)	1935 (61)	1226 (39)	
Yes	519 (14)	278 (54)	241 (46)	
Program type, n (%)				<.001
Medicaid	1630 (44)	962 (59)	668 (41)	
OAA^d^	736 (20)	543 (74)	193 (26)	
PACE^e^/MLTSS^f^	1314 (36)	708 (54)	606 (46)	
Proxy response, n (%)				≥.99
No	2948 (80)	1773 (60)	1175 (40)	
Yes	732 (20)	440 (60)	292 (40)	

a Pearson chi-square test; Welch 2 sample *t* test

b RUCA: rural-urban commuting area.

c ADRD: Alzheimer disease and related dementias.

d OAA: Older Americans Act.

e PACE: Program of All-Inclusive Care for the Elderly.

f MLTSS: managed long-term services and supports.

### Regression Analysis of Telehealth Use by SEIPS Domains

Table 2 presents the full logistic regression model results, with model estimates reported as odds ratios (ORs) and 95% CIs for telehealth use versus no telehealth use. Several significant associations between the covariates and telehealth use emerged from the analysis. Results from the sensitivity analysis using multiple imputation were consistent with those of the complete-case analysis, indicating that missing data did not substantially impact the observed associations.

**Table 2.** Results of the logistic regression model using complete-case analysis for telehealth use. Values are odds ratios (OR) and 95% CI for telehealth use versus no telehealth use.

Characteristic	Participants, n	OR^a^ (95% CI)
Age (years)		
65-74	1783	—^b^
75-84	1238	0.72 (0.62-0.85)^c^
≥85	659	0.66 (0.53-0.81)^c^
Sex		
Female	2667	—
Male	1013	0.80 (0.68-0.94)^d^
Race/ethnicity		
White	1929	—
Black	858	0.72 (0.59-0.87)^c^
Hispanic or Latino	443	1.04 (0.77-1.39)
Multiple race/ethnicity	450	0.95 (0.76-1.20)
Zip code RUCA^e^		
Metropolitan	2660	—
Micropolitan	528	0.75 (0.60-0.93)^d^
Small town	273	0.67 (0.50-0.90)^d^
Rural	219	0.72 (0.52-1.00)^f^
Internet access		
No or sometimes	1536	—
Yes	2144	2.51 (2.15-2.92)^c^
Self-perceived overall health		
Poor	604	—
Fair	1519	0.91 (0.75-1.12)
Good	1176	0.85 (0.69-1.05)
Very good	306	0.89 (0.65-1.20)
Excellent	75	1.36 (0.81-2.27)
Medical transportation access		
No or sometimes	285	—
Yes	3395	1.15 (0.88-1.51)
Lives alone		
No	1937	—
Yes	1743	1.02 (0.88-1.19)
Number of known non-ADRD^g^ diagnoses (categorical)		
0	166	—
1	526	1.26 (0.84-1.90)
More than 1	2988	1.49 (1.02-2.17)^f^
Known ADRD diagnosis		
No	3161	—
Yes	519	1.33 (1.07-1.66)^f^
Program type		
Medicaid	1630	—
OAA^h^	736	0.67 (0.47-0.98)^f^
PACE^i^/MLTSS^j^	1314	0.99 (0.74-1.32)
Proxy response		
No	2948	—
Yes	732	1.06 (0.86-1.30)

a OR: odds ratio.

b Not applicable.

c *P*<.001.

d *P*<.01.

e RUCA: rural-urban commuting area.

f *P*<.05.

g ADRD: Alzheimer disease and related dementias.

h OAA: Older Americans Act.

i PACE: Program of All-Inclusive Care for the Elderly.

j MLTSS: managed long-term services and supports.

Person factors showed several notable associations. Among older recipients of publicly funded HCBS, age was significantly associated with telehealth use, with older age associated with lower odds of telehealth use. Using adults aged 65-74 years as the reference group, those aged 75-84 years had an OR of 0.72 (95% CI 0.62‐0.85), and those aged 85 years and older had an OR of 0.66 (95% CI 0.53‐0.81). Sex was also significantly associated with telehealth use, with males having significantly lower odds of telehealth use relative to females (OR 0.80, 95% CI 0.68‐0.94). Racial differences were observed as well: Black participants had significantly lower odds of using telehealth compared to White participants (OR 0.72, 95% CI 0.59‐0.87), whereas Hispanic or Latino participants and those from other racial backgrounds had similar odds of telehealth use relative to White participants. Additionally, individuals with more than one known non-ADRD diagnosis had higher odds of using telehealth compared to those with no known diagnoses (OR 1.49, 95% CI 1.02‐2.17). Those with a recorded ADRD diagnosis also had higher odds of telehealth use compared to individuals without an ADRD diagnosis (OR 1.33, 95% CI 1.07‐1.66).

Technology and environmental factors were also associated with telehealth use. Individuals living in nonmetropolitan areas had lower odds of telehealth use compared to those in metropolitan areas (micropolitan: OR 0.75, 95% CI 0.60‐0.93; small town: OR 0.67, 95% CI 0.50‐0.90; rural: OR 0.72, 95% CI 0.52‐1.00), although the rural estimate should be interpreted with caution because the upper limit of its CI approaches the null value. Internet access was strongly associated with telehealth use, with individuals reporting internet access exhibiting more than twice the odds of telehealth use compared with those with no or intermittent access (OR 2.51, 95% CI 2.15‐2.92).

Organizational factors—specifically HCBS program type—were associated with differences in telehealth use. Participants receiving services through OAA showed lower odds of telehealth use compared with those enrolled in Medicaid (OR 0.67, 95% CI 0.47‐0.98), while those receiving PACE/MLTSS had similar odds of telehealth use as Medicaid recipients.

No significant associations were observed between telehealth use and self-perceived overall health, medical transportation access, living alone, and response type (self vs proxy response).

### Tools and Technology: Sociodemographic Correlates of Internet Access

Table 3 displays the frequencies of reported internet access, along with the *P* value from the omnibus chi-square test for each sociodemographic characteristic.

**Table 3.** Bivariate analyses of sociodemographic factors and internet access using Pearson chi-square test.

Characteristic	Internet access		*P* value^a^
	No or sometimes (n=1536)	Yes (n=2144)	
Age (years), n (%)			<.001
65-74	606 (34)	1177 (66)	
75-84	571 (46)	667 (54)	
≥85	359 (54)	300 (46)	
Sex, n (%)			.01
Female	1080 (40)	1587 (60)	
Male	456 (45)	557 (55)	
Race/ethnicity, n (%)			<.001
White	749 (39)	1180 (61)	
Black	370 (43)	488 (57)	
Hispanic or Latino	266 (60)	177 (40)	
Multiple race/ethnicity	151 (34)	299 (66)	
Zip code RUCA^b^, n (%)			.06
Metropolitan	1076 (40)	1584 (60)	
Micropolitan	231 (44)	297 (56)	
Small town	128 (47)	145 (53)	
Rural	101 (46)	118 (54)	

a Pearson chi-square test.

b RUCA: rural-urban commuting area.

We conducted bivariate analyses to examine how person-level and environmental factors were associated with internet access, a key technology resource for telehealth. Our analysis revealed significant associations between internet access and age (*P*<.001), sex (*P*=.01), and race/ethnicity (*P*<.001). Participants aged 75-84 years and those aged 85 years and older were less likely to report internet access (667/1238, 54% and 300/659, 46%, respectively) than those aged 65-74 years (1177/1783, 66%). Male participants were also less likely than female participants to report having internet access (557/1013, 55% vs 1587/2667, 60%). Among racial and ethnic groups, Black and Hispanic or Latino participants (488/858, 57% and 177/443, 40%, respectively) were less likely to report internet access than White participants (1180/1929, 61%) and individuals from other racial backgrounds (66%, 299/450). No statistically significant association was found between zip code RUCA classification and internet access.

## Discussion

### Principal Findings

Guided by the SEIPS 3.0 framework, this study is, to our knowledge, the first to report differences in telehealth use among older HCBS recipients through a human factors lens. The SEIPS framework illustrates how health care outcomes emerge not from any single factor, but from the alignment—or misalignment—of people, technologies, organizations, and environments across the broader health care system and the patient journey within it. Applying this lens offers a systems-level perspective for identifying actionable system-level factors that may underlie variation in telehealth use.

The results of this study provide preliminary insight into the multiple system-level elements that may influence who is—and is not—being reached by telehealth. Importantly, the observed associations reflect estimates from a regression model that accounted for all included covariates, estimating each factor’s association with telehealth use while adjusting for potentially confounding influences. These adjusted findings provide a clearer view of the factors that may enable or impede telehealth among older HCBS recipients.

Understanding these dynamics is particularly important as telehealth continues to expand, especially among older adults—a population recognized as a priority group for telehealth services due to their complex health needs and challenges [18,19,32,43-45]. Older adults—particularly those receiving publicly funded HCBS—are vulnerable to mobility issues, transportation barriers, chronic conditions, and poverty that make it challenging to access in-person care [46-51]. Health care organizations and policymakers have emphasized the importance of telehealth in geriatric populations for facilitating care access, improving health outcomes, reducing costs, and ultimately promoting health equity [15,52-55].

Taken together, our findings underscore the value of examining telehealth use as a product of multiple factors operating within the broader health care system. In the following sections, we interpret our findings through the SEIPS 3.0 framework, organized by person, tools and technology, environment, and organizational factors.

### Person Factors

In this study, age, sex, race/ethnicity, number of non-ADRD diagnoses, and the presence of an ADRD diagnosis were associated with differences in telehealth use among older HCBS recipients. It is important to note that compared to the broader population of community-dwelling older adults, HCBS recipients generally have more complex care needs and experience social and financial vulnerability. Consequently, the findings presented here extend prior evidence to older adults receiving HCBS.

Age was inversely associated with telehealth use, with individuals aged 85 years and older exhibiting the lowest odds of using telehealth. This finding is consistent with research demonstrating that older adults face a variety of barriers to adopting new technologies, including low digital literacy, technology-related anxiety, limited access to support, and health-related functional limitations [56-58]. Recent evidence demonstrates that these barriers hinder telehealth adoption in older adults [34] but have a more pronounced impact on those of more advanced age [59]. Consistent with our findings, several studies have similarly shown that, within older adult populations, telehealth users tend to be younger than nonusers [60,61].

We also observed sex differences in telehealth use, with males having significantly lower odds of using telehealth than females. This result aligns with other studies demonstrating that older females are more likely to use telehealth than their male counterparts [62-64]. The reasons for these differences are uncertain and may reflect broader sex differences in health care needs and use patterns among older adults [65,66], specifically those receiving HCBS [67,68]. Further research is needed to examine and explain sex differences in telehealth use among older recipients of publicly funded HCBS.

Importantly, our results revealed racial differences in telehealth use. In our sample of older HCBS recipients, Black older adults had significantly lower odds of using telehealth compared to White older adults, consistent with other studies showing lower telehealth use among Black older adults [33,69,70]. This finding may reflect systemic and structural barriers to telehealth access, including pervasive racial and ethnic disparities in access to technology and broadband internet, a relationship further supported by our bivariate analyses showing differences in internet access by race and ethnicity [71-73].

Beyond sociodemographic characteristics, HCBS recipients with more than one non-ADRD diagnosis had significantly higher odds of using telehealth compared to those with no known non-ADRD diagnoses. This finding aligns with prior research showing that individuals with multimorbidity—defined as the presence of 2 or more chronic conditions—are more likely to use telehealth than those with fewer health concerns [74,75]. Multimorbidity often requires ongoing communication with multiple health care providers, making telehealth a valuable option to reduce the burden of frequent in-person visits. Our results reinforce geriatric care recommendations emphasizing telehealth’s potential to support older adults with chronic health conditions [45], particularly those who are homebound or have difficulty traveling to medical appointments [76]. This may be especially important for older HCBS recipients with multimorbidity, whose HCBS eligibility reflects high care needs and increased vulnerability to systemic and health-related barriers that limit access to in-person care. Future research should examine how telehealth can be integrated into the care of older HCBS recipients with multiple health conditions and how programmatic or policy changes could promote its use in this population.

Older HCBS recipients with a documented ADRD diagnosis had slightly higher odds of telehealth use than those without an ADRD diagnosis. This finding aligns with prior research showing that telehealth is generally well received and shows promise for people with ADRD and their caregivers [77-79]. For people living with dementia and their caregivers, telehealth can reduce the burden of travel to in-person health care appointments and facilitate more frequent assessments and follow-up care with health care providers [79]. Our results reinforce telehealth’s potential value for older HCBS recipients with ADRD while also pointing to opportunities to expand its reach in this population. In our sample, only 46% (241/519) of older HCBS recipients with ADRD reported telehealth use—suggesting that while these individuals were slightly more likely to use telehealth than their counterparts, there remains an opportunity to increase uptake. Future research should explore how telehealth services can meet the unique needs of older HCBS recipients with ADRD.

Finally, several person factors were not significantly associated with telehealth use, including self-perceived overall health, living alone, and whether survey responses were provided by the participant or a proxy. These null findings suggest that, within this population, telehealth use may be more strongly patterned by sociodemographic characteristics and objective health indicators, such as documented diagnoses, rather than by subjective health perceptions or cohabitation status. Viewed together, our findings show that person-level characteristics and capabilities—core components of the SEIPS framework—may influence telehealth use among older adults receiving publicly funded HCBS.

### Tools and Technology

Disparities in technology use—including telehealth—are often linked to gaps in broadband internet availability for vulnerable and marginalized older adults [80]. Our results underscore this relationship, revealing a strong association between internet access and telehealth use. In our sample, older adults with internet access had more than twice the odds of using telehealth compared to those with no or intermittent access. This finding aligns with prior research emphasizing the critical role of internet access in enabling telehealth use [28,81,82].

Our findings support the hypothesis that internet access functions as a core technological tool connecting people to health care systems. When absent or unreliable, the broader system’s capacity to deliver care through telehealth may be compromised. As telehealth continues to expand, increasing broadband internet access is essential to promote equitable access to telehealth services, especially for medically, socially, and financially vulnerable older adults [83]. Evidence suggests that state-level funding programs increase broadband availability, highlighting the potential impact of policy interventions on improving internet access [84]. Conversely, municipal and cooperative restrictions have been shown to decrease broadband availability, underscoring the role of regulatory barriers in limiting connectivity, such as state policies that restrict municipalities and electric cooperatives from providing broadband service—often justified as preventing publicly subsidized competition with private internet providers [84]. Targeted policy efforts, such as state and federal initiatives subsidizing broadband internet for low-income and rural populations, could facilitate more equitable telehealth access for vulnerable older adults.

Consistent with patterns observed in the person factors domain, internet access varied significantly by age, sex, and race/ethnicity. Older adults of more advanced age were less likely to have internet access than those aged 65-74 years, which may help explain the lower odds of telehealth use observed in this group. Additionally, males were less likely than females to report having internet access, aligning with our finding of lower odds of telehealth use among male participants. A prior review found mixed evidence regarding sex differences in internet use among older adults, with some studies reporting no sex effect and others showing higher internet access among older men than women [85]. Our findings differ from these results, possibly due to our focus on older recipients of publicly funded HCBS, a population disproportionately composed of women with care needs experiencing social and financial vulnerability [68]. Additional research is needed to characterize age- and sex-related patterns of internet access in this population and their relationship to telehealth use.

Our results highlight differences in internet access by race and ethnicity. In our sample of vulnerable older adults, Black and Hispanic or Latino individuals were less likely to report having internet access than White individuals and those from other racial backgrounds. This finding may help explain why Black individuals had lower odds of telehealth use than their White counterparts in our regression analysis. Interestingly, we found that Hispanic or Latino older adults were less likely to have internet access; however, their odds of telehealth use were comparable to White individuals. These results suggest differing impacts of internet access on telehealth use but generally align with extensive research documenting racial and ethnic disparities in internet access and use [26,71-73]. Addressing these gaps may require targeted programs that expand internet access among minoritized older adults, including those receiving publicly funded HCBS. Such initiatives could serve as precursors to telehealth use by ensuring vulnerable older adults have the technological tools they need to use telehealth.

Unexpectedly, internet access was not significantly associated with zip code RUCA classification. This result contrasts with extensive literature showing lower rates of broadband internet availability and access in rural communities compared to metropolitan areas [86-88]. Furthermore, this finding was unexpected given that in this sample, older HCBS recipients in nonmetropolitan settings demonstrated lower odds of telehealth use compared to those in metropolitan areas. The lack of association between internet access and zip code RUCA may reflect the smaller sample size of rural participants in our analyses. Future research should focus on characterizing internet access and digital disparities among older HCBS recipients, particularly those in traditionally underserved rural areas. Identifying and targeting specific communities with limited broadband internet access could help address gaps in telehealth use by ensuring older adults have access to foundational technology required for telehealth uptake.

### Environmental Factors

We found that older adults residing in nonmetropolitan areas had lower odds of using telehealth compared to those in metropolitan areas. Our estimate for rural residents should be interpreted with caution, as the CI included the null value. Likewise, bivariate analyses showed no significant association between zip code RUCA classification and internet access. Together, these findings suggest that, within this sample, rurality alone may not capture the full range of environmental mechanisms shaping telehealth use. Nevertheless, rural older adults remain a population for whom telehealth is viewed as particularly beneficial, given the limited availability of specialty care in rural areas and the challenges of traveling long distances to receive services [89]. Although extensive prior evidence shows that rural older adults experience a persistent digital divide—including lower broadband availability, reduced device access, and lower rates of technology adoption compared to metropolitan peers [90-93]—our findings suggest that rurality alone may not directly translate into lower telehealth use within the HCBS population. This may indicate that other system components, such as person, technology, and organizational factors, help compensate for structural challenges based on environmental context. In this study, the absence of a strong rural effect could reflect offsetting influences—such as targeted initiatives to expand or prioritize telehealth access in local areas. This underscores the complexity of telehealth use as an outcome shaped by multiple system elements rather than a single environmental barrier.

Access to medical transportation was not significantly associated with telehealth use. This may reflect that transportation barriers do not clearly determine whether an older HCBS recipient uses telehealth as an alternative to in-person appointments. However, transportation remains an important environmental resource within the broader health care system, likely interacting with person and organizational factors to influence how older adults access and coordinate care. Taken together, the weak and/or null associations for environmental factors in this study suggest that other system variables may play a larger role in shaping telehealth access.

### Organizational Factors

Older HCBS recipients enrolled in OAA had lower odds of telehealth use compared to those enrolled in Medicaid. This difference may reflect underlying organizational differences in how these programs connect participants to the health care system. As mentioned previously, OAA programs, administered by local AAAs, primarily provide supportive services such as home-delivered meals, transportation, and referrals rather than direct medical care benefits. While these services are vital for many older adults, they do not directly address or translate to formal health care access. At most, OAA services are a “bridge” that supports better care access and coordination [94]. However, variability in local AAA resources and service delivery makes it difficult to draw generalizations about the relationship between OAA services and health care access. From these data, we also cannot determine whether OAA recipients differ meaningfully from other HCBS participants in their care needs, which could also contribute to differences in telehealth use. This uncertainty underscores how multiple system elements—such as person factors (eg, health needs) and organizational interfaces—may work together to shape telehealth use. Based on our findings, it is plausible that OAA programs provide a weaker interface between the person and the medical care system, offering essential social support but less integration with health care delivery pathways, which may result in lower telehealth use.

By contrast, Medicaid, PACE, and MLTSS programs are structured to deliver comprehensive health care with low out-of-pocket costs to eligible older adults, often including telehealth as a covered option. These organizational systems offer a stronger interface between the person and the health care system. Consequently, individuals enrolled in these programs may have clearer pathways to telehealth services compared to those enrolled in OAA.

These findings highlight the importance of considering HCBS recipients’ organizational interfaces with the health care system and how they may offer or shape opportunities for telehealth use. Further research is needed to explore telehealth access among older HCBS recipients across different program types. Understanding these organizational dynamics is essential for designing system-level interventions that align HCBS program structures with the evolving role of telehealth.

### Strengths and Limitations

Guided by the SEIPS 3.0 framework, this study leverages a unique dataset to examine factors associated with telehealth use among older adults receiving HCBS. This hard-to-reach population offers valuable insights into telehealth use among socially and financially vulnerable older adults, who are considered likely to benefit from these services [14-17]. However, the NCI-AD dataset has limitations, including a lack of information on the type and frequency of telehealth visits, preference for in-person versus telehealth care, telehealth satisfaction, and future intentions to use telehealth. Data regarding reasons for telehealth nonuse are also unavailable. In addition, the dataset does not capture whether participants were explicitly offered telehealth by their providers or payers, which may introduce variability in access opportunities that is not reflected in our analyses. Consequently, we could not analyze more nuanced aspects of telehealth use and nonuse within this sample. Additionally, the cross-sectional nature of the NCI-AD data limits the ability to draw causal inferences. Recall bias may also affect the accuracy of responses, as participants may not accurately report their telehealth and other health experiences. Furthermore, because the dataset primarily captures older adults receiving HCBS, our findings are most applicable to community-dwelling older adults and may not generalize to those residing in congregate settings. Future research should use longitudinal designs to better understand telehealth use and to examine how interactions across SEIPS components influence telehealth access, use, and effectiveness among older HCBS recipients and older adults more broadly.

### Conclusion

This study uses the SEIPS 3.0 human factors framework to examine differences in telehealth use among older recipients of HCBS living at home, a socially and financially vulnerable population that may benefit greatly from telehealth services in reducing or removing barriers to in-person health care. Our results show that among older HCBS recipients, older individuals, males, those living in nonmetropolitan areas, Black individuals (relative to White), and OAA recipients (relative to those receiving Medicaid) had lower odds of using telehealth. Individuals with multiple diagnoses and those with a documented ADRD diagnosis had higher odds of telehealth use. Notably, internet access was strongly associated with telehealth use and, in bivariate analyses, was associated with similar sociodemographic factors as those linked to telehealth use, emphasizing the need for expanded broadband internet access to promote equitable telehealth use among vulnerable older adults. Viewed through the SEIPS model, the findings reveal differences in telehealth use as an outcome emerging from multiple system elements rather than any single determinant. Future research should identify and address the potential mechanisms underlying these differences to ensure telehealth fulfills its potential to improve access to care and promote health equity for older Americans.

## Abbreviations:

AAAs: Area Agencies on Aging
ADRD: Alzheimer disease and related dementias
HCBS: home- and community-based services
*ICD-10*: *International Statistical Classification of Diseases, 10th Revision*
MLTSS: managed long-term services and supports
NCI-AD: National Core Indicators–Aging and Disabilities
OAA: Older Americans Act
OR: odds ratio
PACE: Program of All-Inclusive Care for the Elderly
RUCA: rural-urban commuting area
SEIPS: Systems Engineering Initiative for Patient Safety
